# Flux-Based Formulation Development—A Proof of Concept Study

**DOI:** 10.1208/s12248-021-00668-9

**Published:** 2022-01-05

**Authors:** Szabina Kádár, Petra Tőzsér, Brigitta Nagy, Attila Farkas, Zsombor K. Nagy, Oksana Tsinman, Konstantin Tsinman, Dóra Csicsák, Gergely Völgyi, Krisztina Takács-Novák, Enikő Borbás, Bálint Sinkó

**Affiliations:** 1 Budapest University of Technology and Economics, 3 Műegyetem rkp, Budapest, 1111 Hungary; 2 Pion Inc., 10 Cook Street, Billerica, Massachusetts 01821 USA; 3 Semmelweis University, 9 Hőgyes Endre Street, Budapest, 1092 Hungary

**Keywords:** absorption, dissolution-permeation, formulation additives, *in vivo* predictive, telmisartan

## Abstract

The work aimed to develop the Absorption Driven Drug Formulation (ADDF) concept, which is a new approach in formulation development to ensure that the drug product meets the expected absorption rate. The concept is built on the solubility-permeability interplay and the rate of supersaturation as the driving force of absorption. This paper presents the first case study using the ADDF concept where not only dissolution and solubility but also permeation of the drug is considered in every step of the formulation development. For that reason, parallel artificial membrane permeability assay (PAMPA) was used for excipient selection, small volume dissolution-permeation apparatus was used for testing amorphous solid dispersions (ASDs), and large volume dissolution-permeation tests were carried out to characterize the final dosage forms. The API-excipient interaction studies on PAMPA indicated differences when different fillers or surfactants were studied. These differences were then confirmed with small volume dissolution-permeation assays where the addition of Tween 80 to the ASDs decreased the flux dramatically. Also, the early indication of sorbitol’s advantage over mannitol by PAMPA has been confirmed in the investigation of the final dosage forms by large-scale dissolution-permeation tests. This difference between the fillers was observed *in vivo* as well. The presented case study demonstrated that the ADDF concept opens a new perspective in generic formulation development using fast and cost-effective flux-based screening methods in order to meet the bioequivalence criteria.

**Supplementary Information:**

The online version contains supplementary material available at 10.1208/s12248-021-00668-9.

**Figure:**
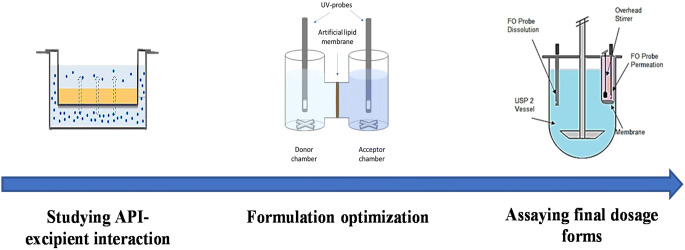
Graphical Abstract

## INTRODUCTION

During the process of formulation development for poorly water-soluble active pharmaceutical ingredients (APIs), multiple excipients and formulation strategies are tested, selected, and optimized based on stability, compatibility, safety, economic considerations, and *in vivo* performance. *In vivo* performance is one of the most challenging parameters to predict and control during the development process and is one of the last criteria to be tested in the form of bioequivalence study or other *in vivo* human clinical trials. A method for rapidly predicting *in vivo* performance throughout all steps of the development process is highly desired as all changes to the composition or the structure can affect the performance. Accordingly any alteration to the formulation for manufacturability, stability, or other reasons potentially invalidates former predictions. According to the USP guideline, dissolution tests should be performed to predict the bioavailability of the drug [1, 2]. The *in vitro* dissolution tests may overestimate the *in vivo* precipitation of drugs as the sink condition created by continuous absorption through the lipophilic membranes is not simulated by the simple dissolution apparatus. Therefore simultaneous dissolution-permeability tests may give better *in vivo* predictions when studying precipitating systems [3–6]. In the recent years, many important studies were published about the correlation between solubility and permeability [7]. The so-called solubility-permeability interplay, as illustrated through *in vitro* and *in vivo* studies, indicate that formulation-associated changes in drug solubility and permeability are inversely proportion when solubility enhancement occurs through an alteration of drug thermodynamic solubility (e.g., via the addition of cyclodextrins, surfactants, and cosolvents) [8–10]. The basis for this relationship has been mathematically described [7].

In case of formulations that lead to supersaturation, such as through the use of amorphous form or lipid-based formulations [11, 12] the thermodynamic solubility of the compound is not altered and the permeability remains unchanged. Formulating the API in form of amorphous solid dispersion (ASD) is a possibility to improve not just its dissolution but also the bioavailability of the API [13]. Electrospinning (ES) is an effective technique for the preparation of ASDs. During the manufacturing, nano-/microfibers are created from a viscous polymer solution containing an API using the driving force of an electrostatic field. This method has several advantages: it is a gentle method for the preparation of solid formulations of the various bioactive agent [14, 15]; it can be a part of continuous manufacturing [16, 17]; and compared to the spray-drying, the amorphous structure is more homogenous and stable as a result of electrostatic forces which lead to more effective solvent evaporation [18, 19]. Polymer additives used for electrospinning are responsible for making the API’s supersaturation last longer in solution, but these excipients generally do not modify the equilibrium crystalline solubility of API. In some cases, however, polymers can affect the thermodynamic solubility of the API, as demonstrated in the literature [20].

It shows the importance of the research area that not only countless studies were carried out in the past, but in recent years, international research collaborations were also established between industry, academia, and even regulatory agencies to improve the prediction of the *in vivo* performance of oral drug products. The OrBiTo project (Oral Bioavailability Tools) was generated across 13 industrial and 14 academic partners. The program aims to increase the understanding of the gastrointestinal absorption process and to develop and validate new, more biorelevant *in vitro* and *in silico* methods, which can result in good *in vivo* prediction [21, 22]. Another research project was funded by the Food and Drug Administration (FDA) to develop an *in vivo*-relevant *in vitro* dissolution testing apparatus [23].

In this work, we present a new approach for generic formulation development which ensures that the drug product meets the expected absorption rate and therefore the bioequivalence criteria. The Absorption Driven Drug Formulation (ADDF) concept investigates and considers the excipient effect on absorption from the excipient selection through the entire development process. In the first step, various types of excipient such as surfactants, polymers, and fillers are investigated to explore the interaction between the API and the excipients using the parallel artificial membrane permeability assay (PAMPA). The generated data are used to select the initial excipients for the preparation of the formulations. In the next step, the effects of individual excipient and formulation process are investigated using small volume flux assay. In the final stage of the development, biorelevant volumes are used on the donor side to investigate and compare final dosage forms.

The aim of this work was to demonstrate that using flux experiments in the formulation development from the excipient selection to the testing of final dosage forms helps the generic drug developers to meet the bioequivalence criteria. Further aim was to show that with the help of high-throughput screening tools like PAMPA and small volume flux assays, fast and cost effective formulation development can be achieved when using the ADDF concept.

For our proof of concept study, we have chosen telmisartan (TEL) as a model drug. TEL is an angiotensin II receptor antagonist and therefore used in the treatment of hypertension [24]. It belongs to the second class of Biopharmaceutical Classification System (BCS), having low solubility and high permeability. The solubility of TEL is extremely low and pH-dependent (practically insoluble between pH 3 and 8) [25, 26]. Micardis, the brand name formulation of TEL, which contains the API in amorphous form, was selected as a reference product in the final stage of flux studies. [27]

## MATERIALS AND METHODS

### Materials

**Figure 1:**
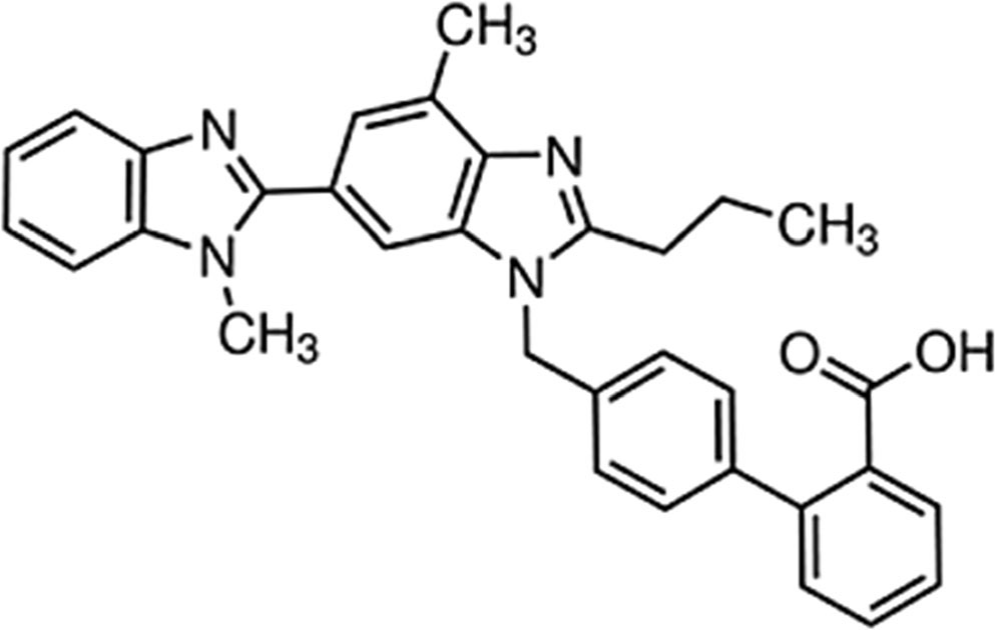
Structure of telmisartan

TEL (514 g/mol, structure shown in Fig. 1), buffer components (NaH_2_PO_4_, NaOH, NaCl, KCl, HCl), sorbitol, sodium dodecyl sulfate (SDS), polyoxyethylene sorbitan monooleat (Tween 80), and *n*-dodecane were purchased from Sigma-Aldrich Co. Llc. (St. Louis, MO, USA). Lactose monohydrate was obtained from Meggle Pharma (Wasserburg, Germany). Reagent grade dichloromethane (DCM) and methanol (MeOH) were ordered from Merck Ltd. (Budapest, Hungary). D-mannitol and (2-Hydroxypropyl)-β-cyclodextrin (HPβCD), with the molar substitution degree of 0.64, were provided by Roquette Freres (Lestrem, France). Hydroxypropylmethylcellulose (HPMC 2910) was obtained from Aqualon, Hercules (Zwijndrecht, The Netherlands). Polyvinylpyrrolidone K30 and K90 (PVP K30 and K90) were received from BASF (Ludwigshafen, Germany). Hydroxypropylmethylcellulose acetate succinate (HPMC-AS) polymer was provided by Ashland Inc. (Wilmington, USA). Prisma^HT^ buffer were obtained from Pion Inc. (Billerica, MA, USA). Simulated intestinal fluid (SIF) powder was purchased from Biorelevant.com (London, UK). Telmisartan brand tablets (Micardis 40 mg API/tablet) were purchased from Boehringer Ingelheim International Gmbh (Ingelheim am Rhein, Germany).

### Flux Experiments

#### PAMPA Method for Evaluating the Effect of Formulation Additives

Each well of the top (donor) compartment of 96-well STIRWELL™ PAMPA sandwich (Pion Inc., Billerica MA, USA) was coated with 4 μL of *n*-dodecane. Before forming the sandwich, the bottom (acceptor) plate was prefilled with 180 μL of pH = 7.4 Prisma^HT^ buffer. The donor plate was filled with 200 μL of FaSSIF buffer with pre-dissolved TEL and formulation additives. The resultant sandwich was incubated at 37°C, and both compartments were stirred at 60 μm (ABL) with Gut-Box™ (Pion Inc., Billerica MA, USA). After 30 min, the PAMPA sandwich was separated, and 100 μL of both the donor and acceptor compartments were transferred to UV plates (UV-star micro plate, clear, flat bottom, half area, Greiner Group AG, Kremsmünster, Austria). UV absorption (230–400 nm) was measured with Tecan Infinite M200 (Tecan Group Ltd., Mannedorf, Switzerland) driven by PAMPA Explorer software™ (Pion Inc., Billerica MA, USA). Three parallel measurements were performed, and the data were measured with six replicates on every single plate.

The flux across the membrane was calculated using the following equation:

1 $$ J(t)=\frac{\Delta n}{A\ast \Delta t} $$

where the flux (*J*) of a drug through the membrane is defined as the amount (*n*) of drug crossing a unit area (*A*) perpendicular to its flow per unit time (*t*).

2 $$ {P}_e=\frac{J}{C_{\mathrm{D}}} $$

where the *P*_e_ is the effective permeability of the drug and *C*_D_ is donor concentration.

#### Small Volume Dissolution-Permeation Measurements with MicroFLUX Apparatus

Electrospun formulations of TEL were tested using μFLUX (Pion Inc., Billerica MA, USA) apparatus which consists of a donor and an acceptor chamber separated by an artificial membrane (PVDF, polyvinylidenfluoride, 0.45 μm, 1.54 cm^2^) impregnated with 25 μL *n*-dodecane to form a lipophilic barrier between the donor and acceptor chambers. In case of oral drug delivery, the donor chamber represents the stomach’s condition, while the 18 mL pH 7.4 buffer of acceptor chamber represents the blood circulation. The attempt was started in 16 mL pH 1.6 buffer solution; then after 30 min, media in the dissolution vessel was converted to FaSSIF (pH 6.5) by adding 4 mL of SIF concentrate. Both chambers were stirred at 250 rpm at 37°C to keep the thickness of the unstirred aqueous layer on minimum. In both chambers, the API concentration was followed by immersed UV probes connected to the Rainbow instrument (Pion Inc., Billerica MA, USA). The flux and permeability values were calculated using Eqs. (1) and (2) between 70 and 100 min.

#### Large Volume Dissolution-Permeation Measurements with MacroFLUX Apparatus

Final dosage forms of TEL were tested using MacroFLUX^TM^ (Pion Inc., Billerica MA, USA). The method used for large-volume flux studies was described in a previous publication by Borbas *et al*. [28]. The detailed description of the parameters can be found in the supplement as well. The flux and permeability values were calculated using Eqs. (1) and (2) between 70 and 100 min.

### Kinetic Solubility Measurements

Note that in the context of this manuscript, solubility measured by this method will be called kinetic solubility because the form of the solid phase precipitating during these assays was not characterized. The concentrated (4 mg/mL) stock solution of TEL was prepared using 1 M NaOH solution as a solvent. Ten milliliters of dissolution medium consisting of equilibrated fasted state simulated intestinal fluid (FaSSIF) buffer containing pre-dissolved additives was stirred at 200 rpm and held at 37°C by circulating water through a heating block mounted to a Pion μDISS™ profiler (Pion Inc., Billerica MA, USA). The stock solution was added in 20 μL aliquots to the dissolution medium. 1 M HCl was used to readjust the pH of the media after every addition of the stock solution. During and after addition of the stock solution, UV probes connected to a Pion Rainbow™ Dynamic Dissolution Monitor® system were used to detect changes in UV spectra, thus enabling the detection of precipitation, which provides information about the kinetic solubility limit.

The zero intercept method (ZIM) was implemented in the AuPRO™ software (Pion Inc., Billerica MA, USA), which was considered a suitable method for detecting the solid nanoparticles in solution. It can be noticed that the second derivative spectrum of a particular compound usually crosses the wavelength axis at specific wavelengths that we will call zero intercept method points or ZIM points (*λ*_*ZIM*_). The ZIM points are characteristic of the compound and are independent of the concentration. The second derivative values at *λ*_*ZIM*_ are expected to be zero until the compound is fully dissolved. After a critical concentration, solid particles appear in the solution, and a ZIM point shift can be observed [29–31]. In case of TEL, the ZIM evaluation was carried out at a wavelength of 318 nm.

### Electrospinning

The calculated polymer and TEL amounts were added into 8 mL of the solvent and stirred magnetically (VELP Scientifica, Usmate, Italy) at 600 rpm until the dissolution was complete (Table I). The electrical potential applied on the spinneret electrode (inner diameter 0.8 mm) was 40 kV (NT-65 high voltage DC supply, MA2000 device, Unitronik Ltd, Nagykanizsa, Hungary). A grounded aluminum plate covered with aluminum foil was used as collector (20–30 cm from the spinneret). Polymer solutions were dosed with 5–15mL/h at room temperature (25°C) with an SEP-10S Plus type syringe pump (Aitecs, Vilnius, Lithuania). The compositions of polymer solutions are shown in Table I. Due to the carboxylic group of TEL, it forms sodium salt (TelNa) in the presence of NaOH.

**Table I:** Compositions of the Polymer Solutions

Formulation	API (w/w %)	PVPK30 (w/w %)	PVPK90 (w/w %)	HPMC-AS (w/w %)	Tween 80 (w/w %)	MeOH (mL/g dry matter)	DCM (mL/g dry matter)	1 M NaOH (mL/g dry matter)
TelNa_PVP	94.34	5.03	0.63	0	0	0	0	2.01
TelNa_HPMCAS	94.34	0	0	5.66	0	0	0	2.01
TelNa_PVP + Tween80	93.48	4.99	0.62	0	0.91	0	0	2.01
TelNa_HPMCAS + Tween80	93.48	0	0	5.61	0.91	0	0	2.01
Tel_PVP	15.09	75.47	9.44	0	0	3.77	3.77	0
Tel_HPMCAS	15.09	0	0	84.89	0	3.77	3.77	0

### Preparation of Amorphous TEL

The solvent evaporation technique was used for the preparation of amorphous TEL. One hundred sixty milligrams TEL was dissolved into 8 mL of the solvent (1:1 mixture of MeOH and DCM), and the solution was evaporated at room temperature.

### Tableting

240 ± 5 mg tablet weights were compressed using a Dott Bonapace CPR6 eccentric tableting machine (Dott Bonapace, Italy), equipped with a 10 mm concave punch. For each tablet, the exact tablet weight and compression force were registered individually. The composition of the tablets is shown in Table II.

**Table II:** Composition of the Tablets

Components	Weight (mg)
API	40
NaOH	3.6
Polymer (HPMC-AS/PVP)	2.4
Filler (mannitol/sorbitol)	191.6
Magnesium stearate	2.4
Summa tablet weight	240

## RESULTS

The results of the permeability assays and kinetic solubility measurement are described, followed by the results of the *in vitro* simultaneous dissolution-permeation tests for the formulations of TEL.

### Screening of Formulation Additives Based on PAMPA and Kinetic Solubility

#### PAMPA

At first, an excipient screening on the PAMPA platform was carried out. The excipients of the available TEL formulations and commonly used standard excipients were involved in the investigation of API-excipient interaction. The concentration of the excipients was determined by referring the usual amount of additives in one tablet to 900 mL dissolution media. Due to the experimental differences in the applied doses, the flux data were considered misleading, and the permeability value was used since it is normalized by the donor concentration.

The results in Table III show that in the case of surfactants, significant permeability reduction can be seen compared to the excipient-free case (*p* value less than 0.05). The reference permeability values in different plates showed low standard deviations. Generally, polymers had a slight, non-significant increasing effect compared to the neat API, but in case of HPMC-AS, the increase in permeability was found to be significant. Finally, mixed effects were experienced with fillers, where in the case of sorbitol and lactose monohydrate, a significant increase was detected, while the addition of mannitol did not impact the permeability of TEL.

**Table III:** Results of Permeability Measurements on PAMPA Platform

Type of additive	Additive	Additive concentration (μg/mL)	P_e_ (10^−4^ cm/sec)	SD	Reference P_e_ (10^−4^ cm/sec)	SD	t-test (*p* value)
Surfactants/complexing agents	**SDS**	**850**	**0.83**	**0.02**	**1.16**	**0.07**	**0.00**
	**Tween 80**	**0.45**	**0.86**	**0.02**	**1.16**	**0.07**	**0.00**
	HPβCD	1386	1.21	0.05	1.16	0.07	0.42
Polymers	HMPC 2910	212	1.22	0.06	1.16	0.07	0.60
	PVP K90	212	1.25	0.18	1.16	0.07	0.99
	PVP K30	212	1.31	0.15	1.11	0.07	0.06
	**HPMC-AS**	212	**1.28**	**0.10**	**1.11**	**0.07**	**0.01**
Fillers	**Sorbitol**	**667**	**1.42**	**0.10**	**1.11**	**0.07**	**0.00**
	Mannitol	667	1.23	0.20	1.11	0.07	0.41
	**Lactose monohydrate**	**667**	**1.39**	**0.06**	**1.16**	**0.07**	**0.02**

#### Kinetic Solubility

Table IV demonstrates that in the case of surfactants, a significant kinetic solubility increase was detected as compared to the excipient-free case (*p* value less than 0.05). The polymers did not significantly affect the kinetic solubility of TEL. Finally, it can be seen that mannitol did not significantly influence the solubility, while sorbitol caused a significant decrease in kinetic solubility.

**Table IV:** Results of Kinetic Solubility Measurements

Type of additive	Additive	Additive concentration (μg/mL)	Kinetic solubility (μg/mL)	SD	t-test (*p* value)
	**-**	0	37.27	3.17	-
Surfactants/complexing agents	**SDS**	**850**	**45.87**	**4.80**	**0.02**
	**Tween 80**	**0.45**	**64.27**	**7.30**	**0.01**
	HPβCD	1386	37.50	0.75	0.88
Polymers	HMPC 2910	212	30.90	0.96	0.05
	PVP K90	212	34.37	1.15	0.36
	PVP K30	212	34.70	1.18	0.41
	HPMC-AS	212	34.10	2.70	0.20
Fillers	**Sorbitol**	**667**	**30.73**	**1.76**	**0.03**
	Mannitol	667	39.67	0.31	0.31
	Lactose monohydrate	667	32.80	2.42	0.25

For the later stages of the development, both PVP versions, HPMC-AS, Tween 80, sorbitol, and mannitol were used in the formulation samples after evaluating PAMPA and kinetic solubility results (see the “Discussion” for details).

### Preparation of ASDs via Electrospinning

Since TEL has extremely low solubility in the biorelevant pH range of the small intestine, the first commercial formulation of TEL, Micardis contains the drug in amorphous form [32]. For the same reason, the strategy of preparing an amorphous solid dispersion-based formulation of TEL has been selected for the ADDF project. Electrospinning, an emerging technology, has been applied to produce ASDs. Based on the excipient interaction study, mixture of PVP derivatives and HPMC-AS have been selected as a matrix polymer.

For the electrospinning process, first a TEL containing polymer solution had to be created (Table I). Because of the poor water solubility of the APIs, volatile organic solvents or a mixture of organic solvents are typically used. However, in the case of TEL, it was found that it dissolves extremely well in 1 M NaOH solution; therefore, it can be electrospun from aqueous media with higher productivity than from organic solvents (Table V).

**Table V:** Characteristics of Electrospun TEL Containing Formulations

Formulation	Electrospun sample	Size range (μm)	Yield (g API/h)	XRD characterization
Tel_Na_PVP	Beadless	2.26–3.87	2.25	Amorphous
Tel_Na_HPMC-AS	Mostly beads	0.56–1.57	2.25	Amorphous
Tel_Na_PVP + Tween 80	Beadless	3.15–5.22	4.5	Amorphous
Tel_Na_HPMC-AS+Tween 80	Beaded	0.57–1.20	6.75	Amorphous
Tel_PVP	Beadless	0.62–0.73	0.12	Amorphous
Tel_HPMC-AS	Beaded	2.45–2.98	0.30	Amorphous

For the product to have the appropriate fibrous structure, a mixture of PVP derivatives was used (Table I). The PVP mixture and HPMC-AS were used in the same concentration in the electrospinning solutions. The scanning electron microscopic images of the electrospun samples (see Supplement) confirm the formation of thin fiber structure in the case of the PVP mixture containing formulation, while no fiber formation, only electrospraying, occurred when using HPMC-AS alone (Table V).

Tween 80 was added to optimize surface tension of both polymer solutions [33] which not only helped the formation of fibers but also significantly increased the process yield (Table V).

The amorphization has been confirmed for all the samples by X-ray powder diffraction measurements (see Supplement). Based on the yield of the electrospinning process, only the formulations produced from NaOH solution were further investigated with the small volume flux testing.

### Testing of Formulations with Small Volume Dissolution-Permeation Apparatus

**Figure 2:**
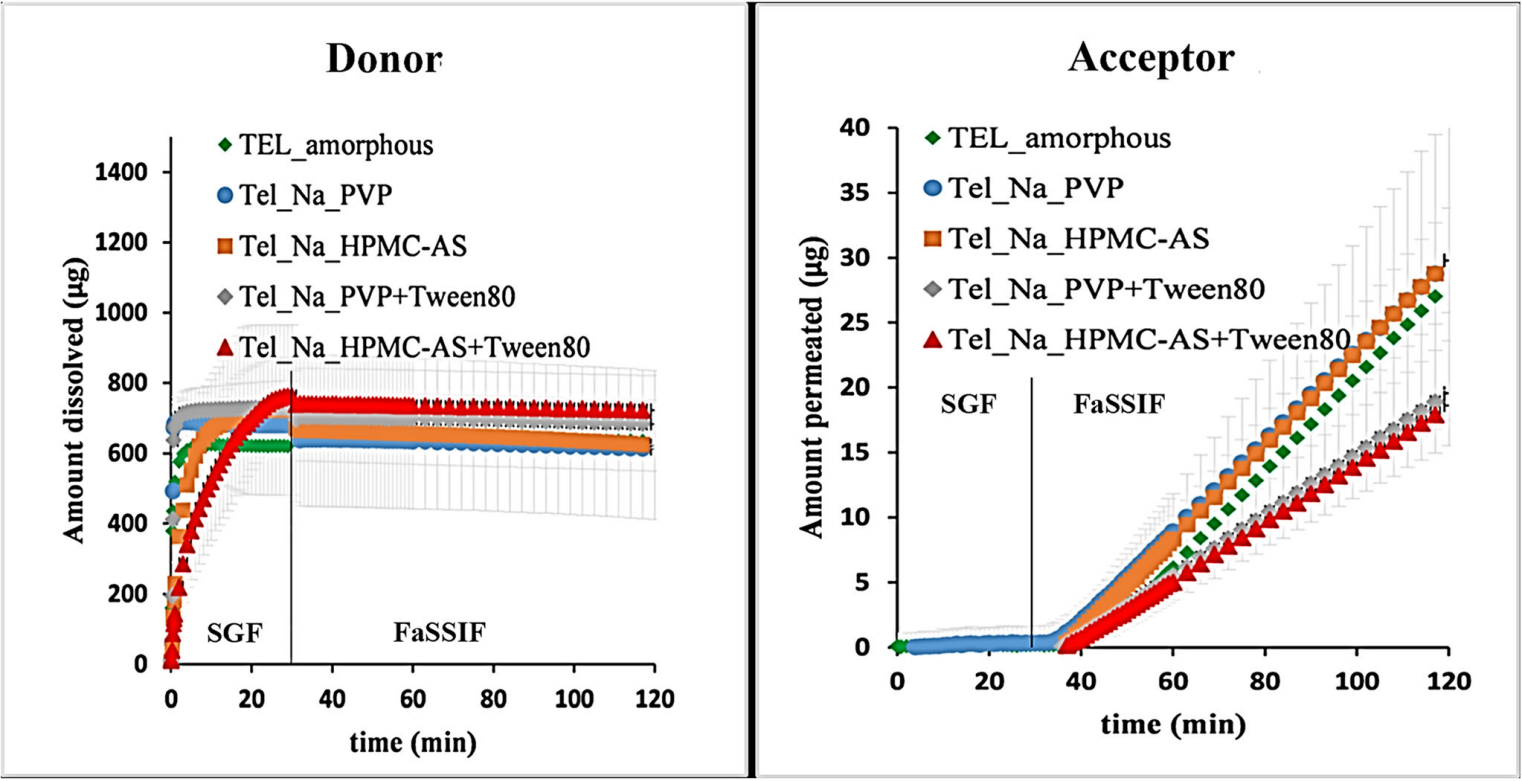
Dissolution in SGF (left) and appearance profile (right) of amorphous telmisartan containing formulations measured by small volume dissolution-permeation apparatus at 37°C

The small volume flux assay has been performed on MicroFLUX setup using a media conversion protocol as described in the “Small Volume Dissolution-Permeation Measurements with MicroFLUX Apparatus” section. The dissolution and appearance profile of the TEL containing ASDs are shown in Fig. 2. As shown on the left graph, all four formulations dissolved completely in simulated gastric fluid (SGF) in the first 30 min. The right-side graph demonstrates that the permeation only started after the media conversion because of the charged state of the API in SGF [28]. To investigate the effect of excipients, formulations were compared to the pure amorphous API. Formulations containing Tween 80 show significantly poorer flux values than did formulations without the surfactant (Table VI). Based on their flux results, only the two electrospun formulations which were prepared without surfactant have been selected and moved to the next stage of final dosage form preparation.

**Table VI:** Permeability Data of Small Scale Dissolution-Permeation Assay of TEL Containing ASDs

Formulation	P_e_ (10^−4^ cm/sec)	SD	t-test (*p* value) compared to amorphous
Tel_amorphous	1.19	0.09	
Tel_Na_PVP	1.21	0.07	0.69
Tel_Na_HPMC-AS	1.21	0.06	0.68
Tel_Na_PVP + Tween 80	**0.74**	**0.12**	**0.01**
Tel_Na_HPMC-AS + Tween 80	**0.58**	**0.05**	**0.00**

The permeability data measured by small volume dissolution-permeation apparatus showed a good agreement with those measured by PAMPA.

### Preparation of Final Dosage Forms via Tableting

Based on the early screening of excipients (PAMPA and kinetic solubility), sorbitol was chosen as a model excipient for lowering solubility and increasing permeability, while mannitol was chosen as an inert tablet filler. Tablets were prepared with these two fillers to confirm the expected difference in the final dosage forms as well. Both ASDs chosen for tableting (Tel_Na_HPMC-AS and Tel_Na_PVP) were mixed and pressed with mannitol and sorbitol separately. Therefore, 4 different tablet versions were made varying the polymer and the filler in the tablets (Table II). These tablets were then assayed with the large volume flux apparatus.

### Testing of Final Dosage Forms with Large Volume Dissolution-Permeation Apparatus

**Figure 3:**
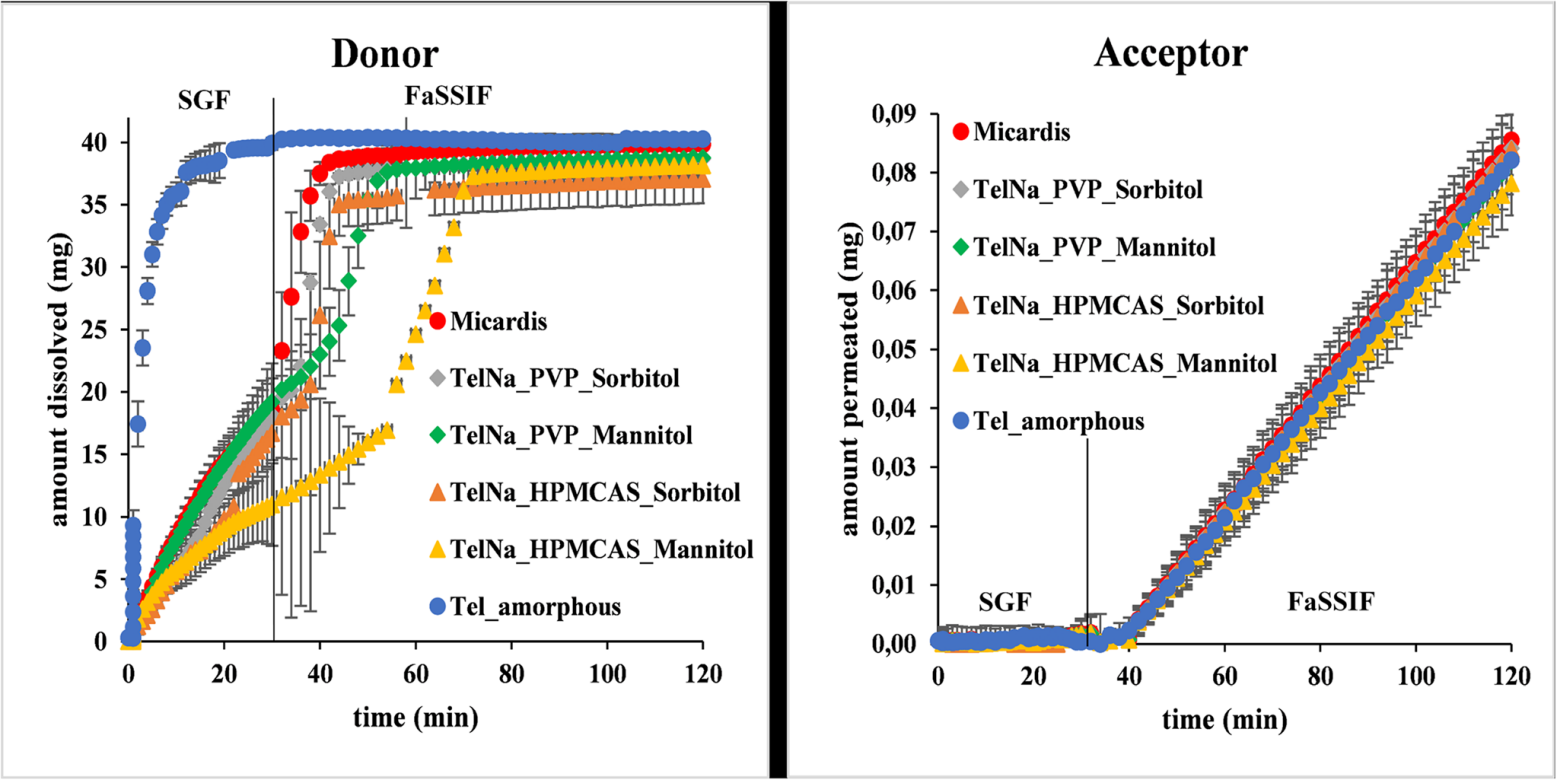
Dissolution in SGF (left) and appearance profile (right) of TEL measured by large volume dissolution-permeation apparatus at 37°C

The MacroFLUX system equipped with *in situ* fiber optic concentration monitoring was used in this final investigation. The same media conversion protocol as in the case of small volume dissolution-permeation measurements was applied in the dissolution vessel [28]. The dissolution and appearance profile of the TEL formulations are shown in Fig. 3. Micardis, the brand formulation, and the pure amorphous drug were used as references. The donor concentration *vs.* time curve on the left side of Fig. 3 shows a delay in disintegration and dissolution for the mannitol containing tablets, but they also reached complete dissolution after about 60–70 min. The permeation started after the media conversion, and the flux was constant in the studied timeframe for all the formulations (Fig. 3, right). The permeability data summarized in Table VII gives a quantitative comparison of the formulations and illustrates how the tablet composition influences the membrane transport of the API. Table VII also shows a comparison of permeability data to the pure amorphous drug and the brand product. It can be seen from the *p* values of Student’s t-tests that 3 formulations out of 4 had significantly higher permeability values than the pure drug.

**Table VII:** Permeability Data of Large-Scale Dissolution-Permeation Assay of TEL Containing Final Dosage Forms

Formulation	P_e_ (10^−4^ cm/sec)	SD	t-test compared to amorphous (*p* value)	t-test compared to Micardis (*p* value)
Micardis	1.16	0.06	**0.01**	
Tel_amorphous	1.03	0.03		**0.01**
Tel_Na_PVP_sorbitol	1.16	0.08	**0.05**	0.97
Tel_Na_PVP_mannitol	1.13	0.03	**0.02**	0.42
Tel_Na_HPMC-AS_sorbitol	1.15	0.07	**0.04**	0.93
Tel_Na_HPMC-AS_mannitol	1.07	0.05	0.21	0.09

The effect of the filler was further investigated; therefore, TEL_PVP and TEL_HPMC-AS samples (previously excluded from the study because of their low yield) were also mixed with mannitol and sorbitol. This resulted in 4 different final dosage forms with high polymer content compared to TEL_Na samples but exactly the same filler content. These final dosage forms were then assayed with large volume dissolution-permeation apparatus (see Supplement). Two ways ANOVA analysis of the permeability results showed that while the quality nor the quantity of the polymer had an effect on the permeability of the API, the type of filler (sorbitol/mannitol) significantly affected the data (*p* = 0.019).

## DISCUSSION

### Excipient Selection Based on PAMPA and Kinetic Solubility Results

Solubility and permeability properties are best evaluated together because of the interplay between the two parameters [34]. In many cases where the compound is poorly water-soluble, the measurements of thermodynamic solubility are very challenging [35]. Since the solubility of crystalline TEL in FaSSIF media is less than 1 μg/mL, the excipient effect could not be studied using the standard saturation shake flask method [36]. Instead, kinetic solubility measurements were carried out using *in situ* UV probes [37]. Surfactants (Tween 80 and SDS) caused a significant increase in solubility (Table IV) and a concomitant decrease in permeability (Table III); sorbitol on the other hand showed a decrease in solubility and therefore also increased the permeability of the API. These results agree well with the literature [10, 34].

### Testing of ASDs with Small Volume Dissolution-Permeation Apparatus

Although Tween 80 was able to improve the productivity of the electrospinning process by 100% in the case of PVP mixture-based formulation and by 200% in the case of HPMC-AS-based formulation (Table V), the use of solubilizing agent also decreased the flux of the formulation by 39–52% (Table VI). This decrease in flux is expected to result in a decrease in oral bioavailability. However, from a product uniformity, stability and quality point of view beadless fibers like PVP mixture samples are preferable to the beaded fibers observed with the HPMC-AS samples (see SEM images in Supplement). Nevertheless, only negligible differences were observed between the permeability of formulations prepared with different polymers (Table VI). For that reason, both PVP mixture and HPMC-AS containing samples, that do not contain surfactant, were selected and moved to the final stage of product development. The decreased permeability observed in the results in case of Tween containing samples agree well with the effects seen in excipient screening with PAMPA method (Tables III–IV) and with rat intestinal permeability studies.

### Testing of Final Dosage Forms with Large Volume Dissolution-Permeation Apparatus

The dissolution profile (Fig. 3) shows that mannitol-containing tablets have slower disintegration and dissolution than the other four formulations. The same phenomenon was shown by Borbas *et al*. when assaying mannitol containing marketed formulations of TEL as compared to the sorbitol containing reference product [28]. The delay is more pronounced in the case of TEL_Na_HPMC-AS_mannitol formulation, probably because of the poor solubility of the polymer in acidic media.

The permeability data shown in Table VII are in good agreement with the early screening results, where the polymers and mannitol slightly but not significantly enhanced the permeability of the API, while in the case of sorbitol, the enhancement was more pronounced. In these 3 cases, the final increase in permeability seems to be the product of the individual effect of each additive. In the case of TEL_Na_HPMC-AS_mannitol, however, it seems that there might be an interaction between the additives, because not only is the disintegration of the tablet is delayed, but also the permeability of TEL from this formulation is lower than expected based on PAMPA results. Micardis contains PVP and sorbitol as additives. When comparing the electrospun samples to the brand tablet, it can be seen that permeability values of TEL_Na_PVP_sorbitol and Micardis are almost identical (Table VII). This result indicates that formulations with identical qualitative composition showed very similar permeability data. Varying the filler of the tablets, however, led to a slight decrease in permeability as compared to the brand product.

With these results, the early indication of sorbitol’s advantage over mannitol by PAMPA has been confirmed in the investigation of the final dosage forms. This difference between mannitol and sorbitol can be observed *in vivo*, as the Cmax of the Actavis (mannitol containing generic product) was found lower than the Cmax of Micardis in the bioequivalence study [32]. As the final dosage forms contain relatively low amount of sugar alcohols, which most likely do not affect the GI transit time [38], the lower Cmax value can rather be explained by the permeability change seen in the *in vitro* setups.

## CONCLUSION

The ADDF concept was created to understand the effect of formulation-related decisions on the flux of the API. This paper presents the first case study using the ADDF concept: not only dissolution and solubility but also permeability of the drug is considered in every step of the formulation development.

Kinetic solubility and PAMPA were used for early excipient screening in order to select excipients or additives which improve the permeability of the API. This high-throughput method could successfully describe the solubility-permeability interplay for TEL with the 10 excipients used in this study.

Small-scale dissolution-permeation apparatus was used for testing TEL containing ASDs. The results showed that while the use of surfactants might be advantageous for the electrospinning process from a productivity point of view, it also significantly decreased the permeability of the API. Therefore, there is a risk that it could lower drug oral bioavailability.

The early indication of sorbitol’s advantage over mannitol by PAMPA has been confirmed in the investigation of the final dosage forms by large-scale dissolution-permeation tests. A difference was also observed *in vivo* between the fillers.

The presented case study showed that the testing of permeability in every step of the development process opens a new perspective of excipient selection and testing of formulations before bioequivalence studies is conducted. The ADDF concept utilizes fast and cost-effective screening methods and therefore may be a helpful new tool for generic drug developers to meet the bioequivalence criteria.

## Supplementary Information

ESM 1 (DOCX 18 kb)

ESM 2 (PNG 1308 kb)

High resolution image (TIF 5697 kb)

ESM 3 (PNG 186 kb)

High resolution image (TIF 1159 kb)
