# Supplementary material for: Flux-Based Formulation Development—A Proof of Concept Study
Source: AAPS J. 2022 Jan 5;24(1):22. doi: 10.1208/s12248-021-00668-9 (PMC8816521; doi:10.1208/s12248-021-00668-9)
Supplement: Supplementary file 1 — (DOCX 18 kb) [file 12248_2021_668_MOESM1_ESM.docx]

Flux based formulation development - a proof of concept study

Szabina Kádár^1^, Petra Tőzsér^1^, Brigitta Nagy^1^, Attila Farkas^1^, Zsombor K. Nagy^1^, Oksana Tsinman^2^, Konstantin Tsinman†^2^, Dóra Csicsák^3^, Gergely Völgyi^3^, Krisztina Takács-Novák^3^, Enikő Borbás*^1^, Bálint Sinkó*^2^

| ^1^ Budapest University of Technology and Economics, 3 Műegyetem rkp, Budapest, 1111, Hungary  ^2^Pion Inc., 10 Cook Street, Billerica, MA 01821, USA  ^3^Semmelweis University, 9 Hőgyes Endre Street, Budapest, 1092, Hungary  *corresponding authors: Bálint Sinkó (email: bsinko@pion-inc.com)  Enikő Borbás (email: eniko.jaksaneborbas@edu.bme.hu  Supplementary Materials |
| --- |

1. Methods

## Large volume dissolution-permeation measurements with MacroFLUX apparatus

Receiver chamber integrated with permeation membrane, overhead stirrer and fiber optic UV probe was inserted in the standard 900 mL vessel of USP 2 apparatus (Erweka DT 126 Dissolution Tester, Heusenstamm Germany). A filter-supported artificial membrane (hydrophobic PVDF, polyvinylidenfluoride, 0.45 μm pore size, 3.8 cm^2^) impregnated with 50 μL of *n*-dodecane was separating the dissolution (donor) compartment from the receiver compartment containing 13 mL of pH 7.4 (Prisma^HT^, Pion Inc.). The experiment began in 850 mL of pH 1.6 buffer simulating gastric conditions (SGF) on the donor side and then after 30 minutes media in the dissolution vessel was converted to fasted state simulated intestinal fluid (FaSSIF V1) (pH 6.5) by adding 212.5 mL of specially formulated concentrate containing SIF powder. Donor stirring was set to 100 rpm, while the receiver stirring was set to 250 rpm to keep the thickness of unstirred water layer on minimum. The integrated fiber-optic UV probes were positioned in the donor and receiver compartments allowing real time concentration monitoring in both chambers. Concentration monitoring was enabled through fiber optic UV probes connected to the Rainbow Dynamic Dissolution Monitor instrument (Pion Inc, Billerica, MA, USA).

## Scanning electron microscopy

The morphology of the samples was investigated with a JEOL JSM-6380LA (JEOL Ltd., Tokyo, Japan) type scanning electron microscope (SEM). Each specimen was fixed by conductive double-sided carbon adhesive tape and coated with gold–palladium alloy before examination. The applied accelerating voltage and working distance were between 15 and 30 kV and 10 and 12 mm, respectively.

## X-ray powder diffraction

X-ray powder diffraction (XRD) patterns of the samples were recorded by means of a PANalytical (Amelo, the Netherlands) X’pert ProMDP X-ray diffractometer using Cu-K" α radiation (1.542 Å) and a Ni filter. The applied voltage was 40 kV, while the current was 30 mA. The samples were analyzed between 4° and 42° 2θ.

1. Results

## Scanning electron microscopy

Scanning electron microscopic (SEM) images of TEL containing electrospun formulations are shown in Fig. S1. The products were observed differences in the structure of the fibers. Changing the polymer concentration can affect the solution viscosity, and higher viscosity favors formation of fibers without beads. The sufficient polymer concentration to enable fiber formation differs for different polymers. The two polymers were used in the same concentration in the electrospinning solution. As can be seen the samples containing PVP have uniform and beadless fibrous structures. However, if the polymer concentration is low in a specific solvent, then pulverization occurs and spheres may be formed, this is shown in the SEM image of TelNa_HPMC-AS. At the boundary of the critical value of the polymers a transition can be observed, so-called fiber-flaws, beads appear next to the fibers this is shown in the SEM image of TelNa_HPMC-AS+ Tween 80.

## X-ray Powder Diffraction

X-ray powder diffraction (XRD) was used to characterize the morphological changes of TEL induced by electrospinning. Fig. S2. shows the diffractograms of the ES samples compared to those of the crystalline API. In the case of the crystalline API (Fig. S2. a), the characteristic peaks of the crystalline drug were clearly observed. Meanwhile, in the case of the formulation (Fig. S2. b-e), no diffraction peaks were seen, only a diffuse background scattering as a proof of the amorphous nature of all the samples.
