# Supplementary figures and images for: Flux-Based Formulation Development—A Proof of Concept Study
Source: AAPS J. 2022 Jan 5;24(1):22. doi: 10.1208/s12248-021-00668-9 (PMC8816521; doi:10.1208/s12248-021-00668-9)

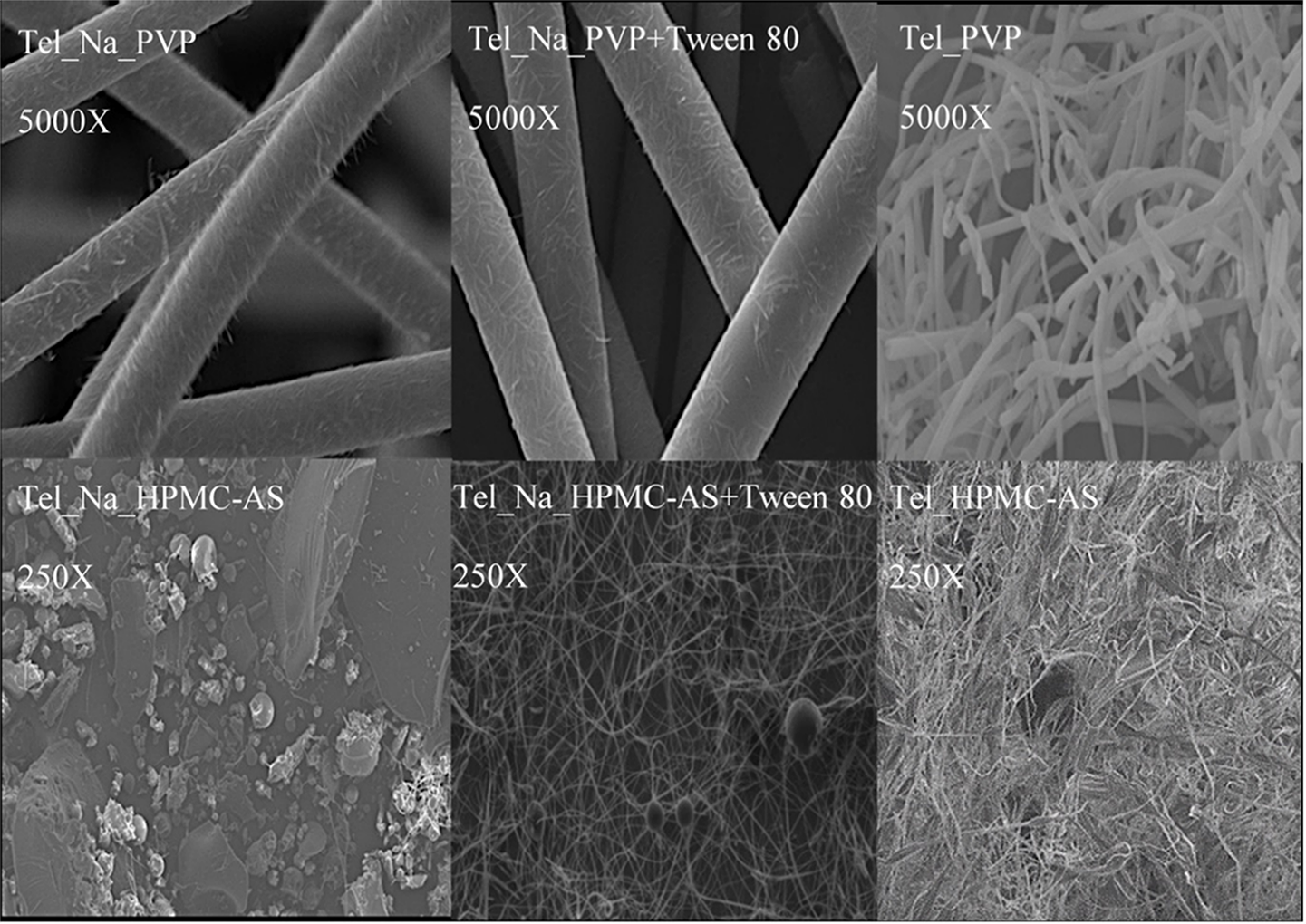

Supplement: Supplementary file 2 — (PNG 1308 kb) [file 12248_2021_668_Fig4_ESM.png]

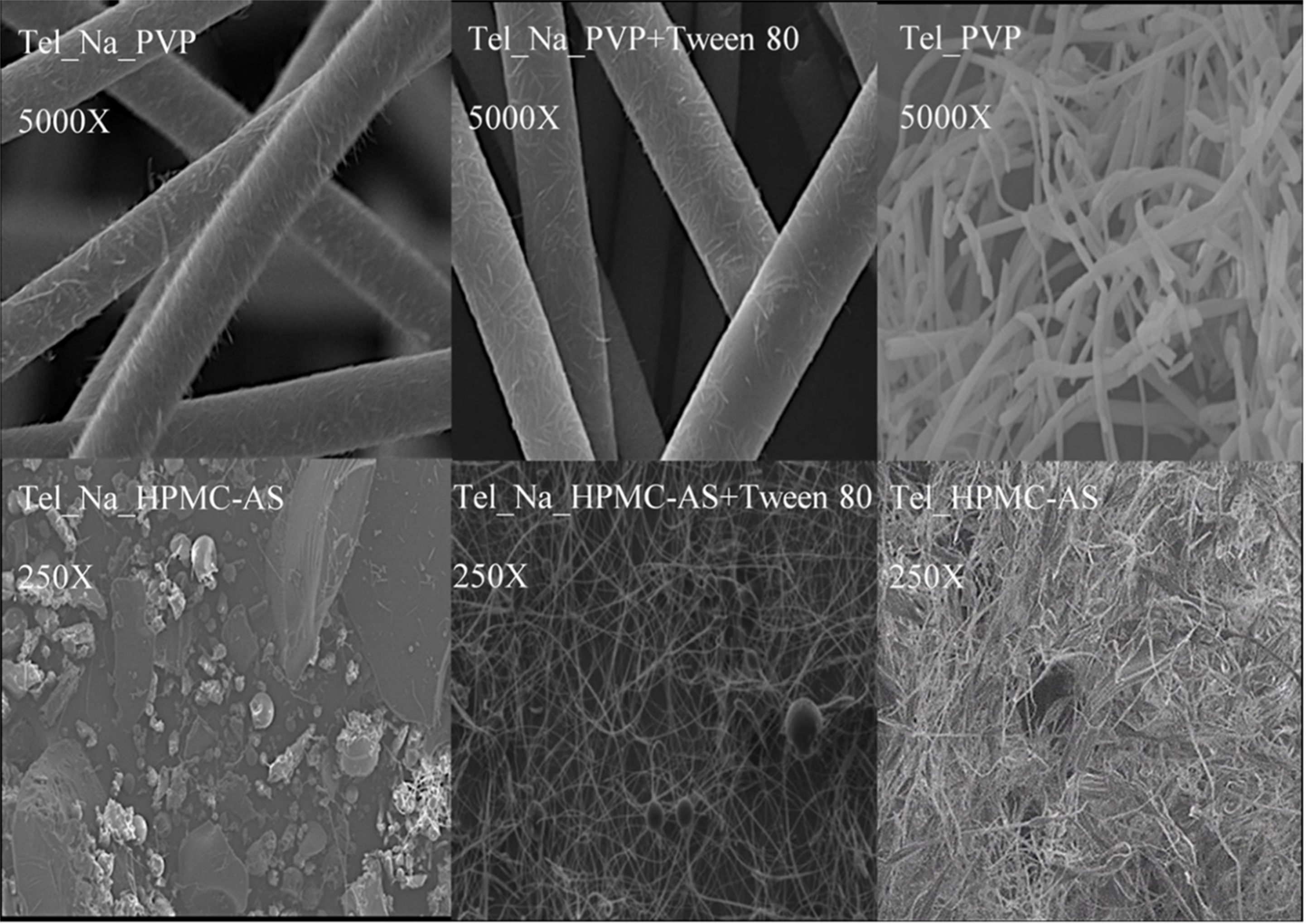

Supplement: Supplementary file 3 — High resolution image (TIF 5697 kb) [file 12248_2021_668_MOESM2_ESM.tif]

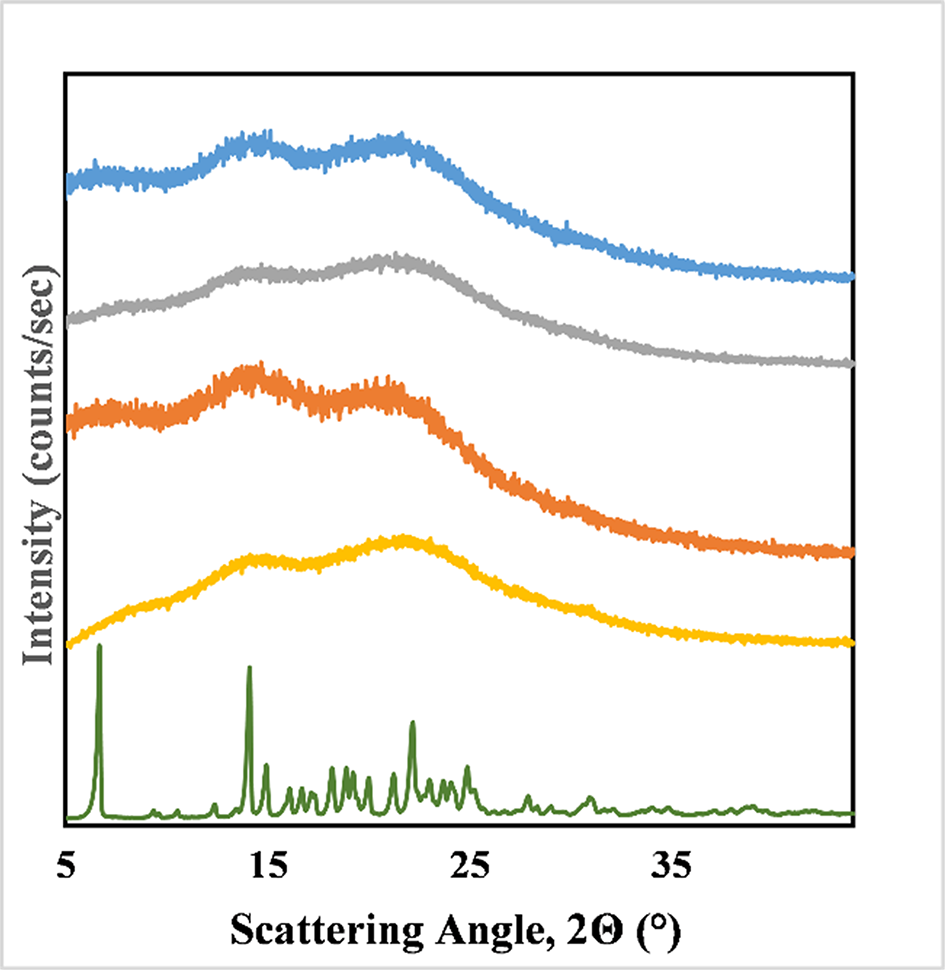

Supplement: Supplementary file 4 — (PNG 186 kb) [file 12248_2021_668_Fig5_ESM.png]

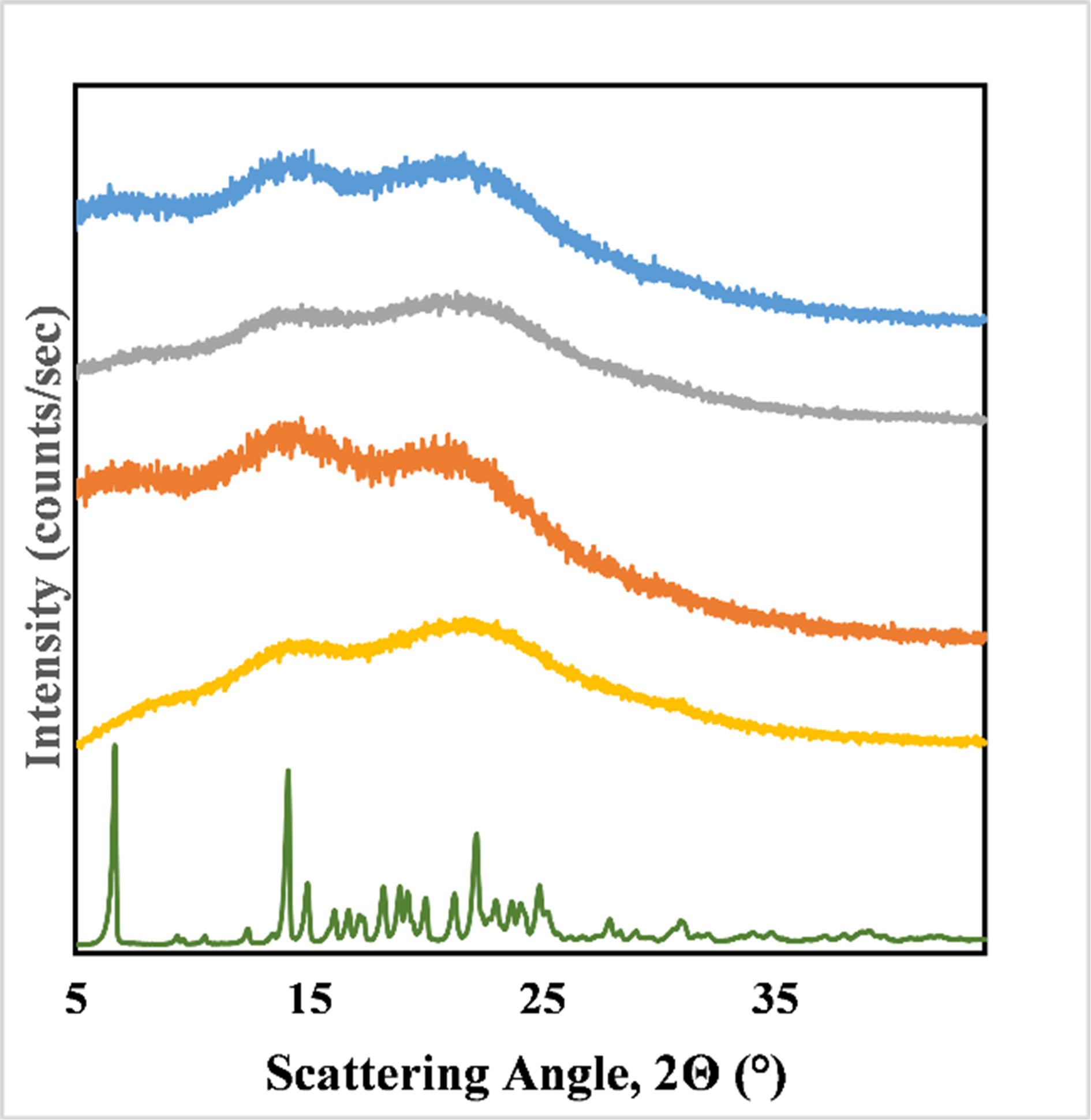

Supplement: Supplementary file 5 — High resolution image (TIF 1159 kb) [file 12248_2021_668_MOESM3_ESM.tif]
